# Effect of type 1 diabetes on the inflammatory response in periodontal disease

**DOI:** 10.3389/fimmu.2025.1683219

**Published:** 2025-11-06

**Authors:** Ana Mendes-Frias, Alexandra Viana da Costa, Filomena Salazar, Ana Braga, Ricardo Silvestre, Marta Relvas

**Affiliations:** 1 Life and Health Sciences Research Institute (ICVS), School of Medicine, University of Minho, Braga, Portugal; 2 ICVS/3B´s-PT Government Associate Laboratory, Braga, Portugal; 3 Associate Laboratory i4HB - Institute for Health and Bioeconomy, University Institute of Health Sciences - CESPU, Gandra, Portugal; 4 UCIBIO - Applied Molecular Biosciences Unit, Toxicologic Pathology Research Laboratory, University Institute of Health Sciences (1H-TOXRUN, IUCS-CESPU), Gandra, Portugal; 5 Department of Medicine and Oral Surgery, University Institute of Health Sciences (IUCS-CESPU), Gandra, Portugal; 6 Oral Pathology and Rehabilitation Research Unit (UNIPRO), University Institute of Health Sciences (IUCS-CESPU), Gandra, Portugal; 7 ALGORITMI Research Center/LASI, University of Minho, Guimarães, Portugal

**Keywords:** type 1 diabetes, periodontal diseases, oral fluids, inflammatory response, cytokine profile

## Abstract

**Introduction:**

Type 1 diabetes mellitus (T1DM) is increasing globally and represents a significant public health concern. Periodontitis affects about 11% of the global population, particularly in its severe forms, and is 1.5 to 2 times more prevalent in individuals with poorly controlled T1DM. Both conditions are multifactorial, chronic, and inflammatory, sharing a bidirectional relationship: T1DM accelerates the onset and progression of periodontitis, while periodontal inflammation worsens glycemic control.

**Methods:**

This observational case-control study included adults with T1DM and metabolically healthy controls, stratified by periodontal status: healthy, gingivitis, or periodontitis. Cytokine profiles were assessed in both saliva and gingival crevicular fluid (GCF) to characterize the oral immune response.

**Results:**

Significant associations were observed between T1DM and both the extent and severity of periodontal disease. T1DM patients with gingivitis exhibited increased bleeding on probing (BOP) and probing pocket depth (PPD), with BOP remaining significantly elevated in those with periodontitis. GCF analysis revealed a dysregulated immune profile in T1DM patients, characterized by elevated IL-1 β, IL-6, IL-8 and IL-17A, and reduced levels of IL-2, IL-4, IL-12p70 and IP-10. The salivary cytokine profile generally mirrored GCF findings, with higher IL-6 and IL-8 concentrations and strong correlations with key pro-inflammatory cytokines.

**Discussion:**

Salivary IL-8 emerged as the most promising biomarker for distinguishing periodontal status in T1DM patients. Overall, these findings highlight the clinical potential of salivary immune profiling as a non-invasive tool for monitoring periodontal inflammation and assessing disease activity in individuals with T1DM.

## Introduction

Type 1 diabetes mellitus (T1DM) is a chronic autoimmune disorder characterized by the destruction of pancreatic β-cells, resulting in insulin deficiency and persistent hyperglycemia (1, 2). Although it typically manifests during childhood or adolescence, T1DM can occur at any age. Affected individuals require lifelong insulin therapy to achieve glycemic control and prevent both acute complications, such as diabetic ketoacidosis, and long−term vascular and neurological sequelae (3, 4). Diabetes remains one of the fastest-growing global health challenges of the 21^st^ century. There are estimated 9.5 million people living with T1D globally in 2025, an 13% increase since 2021, with 1.85 million of these were under 20 years old. In lower-income countries, prevalent cases increased by 20% from 1.8 million in 2021 to 2.1 million in 2025. The projected number of people living with T1D in 2040 is 14.7 million. The largest increase will be in people aged ≥ 20 years (5). In Portugal, T1DM affects an estimated 34,000 individuals, with an annual increase of 2.2% (5, 6). T1DM pathogenesis involves a combination of genetic predisposition and environmental triggers that elicit autoimmune responses, most notably T-cell-mediated β−cell destruction. This immune attack is associated with the release of various inflammatory mediators that further exacerbate β-cell dysfunction and promote apoptosis (7, 8).

Periodontitis is a chronic inflammatory disease initiated by dysbiotic microbial biofilms, leading to progressive destruction of the periodontal tissues and alveolar bone (9). This process is driven by host innate and adaptive immune responses, including dysregulated pro-inflammatory cytokine production and sustained inflammation (10, 11). T1DM and periodontal disease (PD) are interconnected through a bidirectional relationship, primarily mediated by systemic inflammation and the cytokine cascade. In both conditions, hyperglycemia exacerbates periodontal inflammation by promoting systemic pro-inflammatory cytokine release and impairing immune responses (12–14). In turn, periodontal inflammation can impair glycemic control by increasing insulin resistance. While this interaction is well documented in T2DM, it is increasingly recognized in T1DM as well (15).

Patients with T1DM often exhibit more severe PD than non−diabetic individuals, characterized by elevated systemic levels of pro-inflammatory cytokines such as IL−1β, IL−6 and TNFα. These cytokines not only contribute to periodontal tissue destruction but also interfere with insulin signaling in peripheral tissues, thereby worsening metabolic control (15–17). Studies in children and adolescents with T1DM have reported a higher prevalence and severity of gingivitis, with increased risk of progression to more severe periodontitis compared to healthy peers (18, 19).

Despite the considerable focus on pediatric populations, the oral inflammatory profile of adult T1DM patients with PD remains inadequately characterized. Addressing this gap is essential to identify local immune alterations and understand the mechanisms linking systemic metabolic dysfunction to oral inflammation in adults. Saliva and gingival crevicular fluid (GCF) offer non-invasive, readily accessible windows for assessing periodontal and systemic inflammation. Saliva reflects the overall inflammatory state and contains cytokines, matrix metalloproteinases, and other biomarkers, while GCF, collected at the gingival sulcus, provides site-specific information on immune activity and tissue breakdown (20, 21).

In our study, we conducted an observational, analytical case–control study of adults with T1DM and in metabolically healthy controls, stratified by periodontal status (health, gingivitis or periodontitis). We quantified pro−inflammatory and anti−inflammatory cytokines in both saliva and GCF to characterize the local immune responses. This approach enables a more comprehensive understanding of oral-systemic inflammatory interactions and may inform the development of targeted diagnostic and therapeutic strategies.

## Materials and methods

### Study design and ethical approval

This observational, analytical case–control study was conducted at the Endocrinology Departments of two hospital units (Penafiel and Amarante), both affiliated to the Tâmega e Sousa Hospital Center (CHTS). According to the 2023 annual resident estimates by the National Statistics Institute (INE), the CHTS service area includes approximately 510.000 individuals. The present study received ethical approval from the Ethics Committee of CTHS and the University Institute of Health Sciences (IUCS-CESPU). All participants gave written informed consent after a comprehensive explanation of the study’s objectives. The study complies with the ethical principles of the Declaration of Helsinki and follows CONSORT guidelines for conduct and reporting. To ensure measurement reliability and reproducibility of examiners measurement accuracy, two senior periodontists (FS and MR) were calibrated using 10 volunteers assessed twice over a 48-hour interval. Both independently assessed the same subjects to evaluate reproducibility. Intra-examiner correlation coefficients (CCI) for CAL and PD were 0.97 and 0.98, while inter-examiner CCIs were 0.98 for both parameters.

### Study population and clinical assessments

Using a 95% confidence level and a 5% margin of error, a required sample size of 118 patients was calculated from the Endocrinology Department’s population of 170 eligible T1DM patients. However, after accounting for a potential 10% dropout rate and recruitment limitations, the final study cohort consisted of 95 individuals aged between 18 and 70 years. The control group consisted in 95 systemically healthy (non-diabetic) individuals, categorized into three subgroups: healthy, gingivitis, and periodontitis. Exclusion criteria included: pregnancy, history of oral and maxillofacial cancer, prior radiation therapy, other oral mucosal pathologies, periodontal treatment in the past six months; oncological treatment or bone-related medications; hepatic or renal disease, other serious systemic or transmittable diseases; history of alcohol or drug abuse, use of antibiotics or anti-inflammatory drugs in the last 6 months; routine use of oral antiseptics; and presence of implants or orthodontic appliances. The inclusion criteria were aged between 18 to 70 years and at least 18 natural teeth. Anamneses included: gender, age, smoking status (current smoker≥1-year, ex-smoker: quit< 5 years ago, and non-smoker >5 years), and oral hygiene habits such as frequency of toothbrushing and use of dental floss and interdental brush. Periodontal clinical parameters recorded: number of missing teeth; number of mobile teeth, probing pocket depth (PPD), measured as distance from the gingival free-margin from the bottom of the pocket; gingival recession (REC) as the distance from the enamel–cement junction (CEJ) to the free gingival margin, (showing a negative signal whenever the gingival margin is located coronary at the (CEJ); clinical attachment loss (CAL); plaque index (PI) and bleeding on probing (BOP). Measurements were taken at six sites per tooth (mesio-vestibular, vestibular, disto-vestibular, mesio-lingual, lingual, and disto-lingual), using a CPITN 15 periondontal probe (Hu-Friedy Europe, Rotterdam, the Netherlands). Full-mouth periapical radiographs were obtained using the long cone paralleling technique using Rinn holders. Wisdom teeth were excluded from the analysis.

Clinical data collected for consenting diabetic participants included diabetes duration, treatment regimen, glycemic control history, presence of micro- and macrovascular complications, concomitant cardiovascular risk factors, blood pressure, and body mass index (BMI measured in Kg/m2). Glycated hemoglobin (HbA1c) was assessed on the same day at the dental examination. Optimal glycemic control is defined if HbA1c lower than 7%. The insulin regimen is categorized as basal insulin (utilizing solely intermediate-acting or long-acting insulin); basal-plus (basal insulin supplemented with rapid-acting insulin for correction); basal-bolus (basal insulin plus a rapid acting insulin as a prandial plus correction insulin); carbohydrate counting; and premixed insulin.

### Case definition

Periodontal health (healthy controls (HC)) and Periodontal status were defined according to the new consensus of the AAP/EFP (3). Gingivitis was defined as a total percentage of bleeding on probing equal to or greater than 10%, with a probing depth of 3 mm or less. Periodontitis was diagnosed when there was clinical attachment loss (CAL) of 2 mm or more in two or more non-adjacent interproximal sites, or when interproximal CAL was 3 mm or more on the buccal or lingual/palatal surfaces of at least two teeth, accompanied by marginal radiographic bone loss evident on a periapical X-ray. Systemic health effects were also considered, as defined by Tonetti et al. (22). Healthy sites from individuals with no periodontal disease (PD ≤ 3 mm without BOP) were included as healthy controls (HC).

### GCF and saliva collection and preparation

Unstimulated saliva samples were collected using the spitting method, with patients abstaining from oral hygiene, eating, drinking, or chewing gum for at least one hour before collecting. Samples were stored at –80°C until analysis. Before analysis, they were thawed and centrifuged at 6000 g for 10 minutes at 4°C. The resulting supernatants were collected, and aliquots were stored at –80°C for further analysis (15). GCF samples were collected from all participants using eight paper strips (Periopaper^®^, ProFlow, Amityville, NY, USA), placed into the pocket until mild resistance was felt, and left in place for 30s as previously reported (23). Strips contaminated with blood or saliva were discarded. Paper strips were immediately transferred into eppendorf tubes with 300μL sterilized phosphate-buffered saline solution (PBS) and stored at – 80°C until further procedures. Prior to assay, eppendorf tubes were unfrozen and after a 30 minutes elution at room temperature, paper strips were removed and GCF samples were centrifuged at 6000g for 10 minutes at 4°C and, later, aliquots stored at -80°C.

### Cytokine quantification

Cytokine quantification in saliva and GCF samples was performed using the LEGENDplex™ Human Essential Immune Response Panel (13-plex, Ref. 740930, Biolegend, CA, USA), following the manufacturer’s instructions. Cytokine concentrations were expressed in pg/mL, and only values above the detection limit were included in the statistical analysis. The panel quantified 13 key immune response targets: IL-4, IL-2, CXCL10 (IP-10), IL-1β, TNFα, CCL2 (MCP-1), IL-17A, IL-6, IL-10, IFNγ, IL-12p70, CXCL8 (IL-8), and free active TGF-β1. Standards and experimental samples were acquired using an LSR II flow cytometer with FACS Diva software (version 6.1.3; BD Biosciences, USA). Data were analyzed using the LEGENDplex™ Data Analysis Software Suite (Biolegend, CA, USA). The detection limit was 2.44 pg/mL for all cytokines, except IL-1β (2.69 pg/mL), MCP-1 (2.2 pg/mL), IL-17A (3.66 pg/mL), IL-6 (2.2 pg/mL), IL-8 (2.69 pg/mL), and TGF-β1 (5.37 pg/mL). Data reproducibility was ensured, with coefficients of variation between replicates not exceeding 15%.

### Statistical analysis

Statistical analysis was performed using SPSS version 28 software (IBM, New York, NY, USA) and data were plotted using GraphPad Prism version 9 software (San Diego, CA, USA). A two-way ANOVA with mixed-effect model was employed to compare control and diabetic individuals within each periodontal disease group, followed by a Dunnet’s multiple comparisons test. When three groups were compared, the Kruskal–Wallis test was applied to identify statistical differences. For variables that attained global significance, pairwise comparisons were performed using Dunn’s multiple comparisons test. The chi-square test was performed for categorical variables to assess the dependence between variables. Correlations were measured using Spearman’s correlation coefficient. Statistically significant values are indicated as follows: * *p* ≤ 0.05, ** *p* ≤ 0.01, ***p < 0.001 and ****p < 0.0001.

## Results

### Demographic and clinical characterization of the cohort

Sex distribution was identical in the control and T1DM groups, with equal proportions of males and females (**Table 1**). Although smoking habits are known to significantly impact periodontal health, no significant association was found between smoking status and the presence of T1DM in this cohort. Regarding periodontal classification, patients were categorized as healthy, gingivitis, or periodontitis according to the criteria described in the Material and Methods section. As expected, a strong and statistically significant relationship was observed between T1DM and more severe periodontal disease. The control group showed a higher proportion of healthy individuals, while the diabetic group exhibited a greater number of periodontitis cases. A significant association was observed between disease extent and the presence of type I diabetes, with T1DM individuals exhibiting a higher proportion of generalized periodontal disease compared to non-diabetic controls. Age was controlled in this study to ensure comparable groups and to minimize confounding effects, given that type I diabetes is typically diagnosed early in life.

**Table 1:** Demographic characterization of the cohort.

Variable		Group		Statistical analysis		
		Control (n=95)	Diabetic (n=95)	Statistic	*p* value	Effect Size*
Gender, n (%)	Male	62 (33)	62 (33)	0	*p* = 1	0
	Female	33 (17)	33 (17)			
Smoker n (%)	Non smoker	75 (39)	76 (40)	1.05	*p* = 0.59	0.07
	Smoker	20 (11)	19 10)			
Periodontal disease n (%)	Healthy	61 (32)	11 (6)	58.9	*p* < 0.001	0.56
	Gingivitis	20 (11)	33 (17)			
	Periodontitis	14 (7)	51 (27)			
Disease extension, n (%)	Localized	14 (12)	11 (10)	12.9	*p* < 0.001	0.33
	Generalized	18 (15)	73 (63)			
Age, median (IQR)		32 (20)	32 (24)	4425	*p* = 0.82	0.03

*For categorical variables Chi-square test was used to access the dependence of variables; effect size measures (Phi or Cramer’s V to 2x2 comparations or more, respectively) and p-value are reported. For scale variables, Mann-Whitney test was employed; effect size measure (d_Cohen_) and *p*-value are reported. IQR, Inter quartile range; CAL, clinical attachment loss; PI, plaque index; BOP, bleeding on probing, PPD, pocket depth.

Focusing on the T1DM group, a clinical characterization was conducted across the three periodontal disease stages, including clinical features and treatment information (**Table 2**), as well as standard quantitative measures used in the management of T1DM (**Table 3**). No significant associations were found between periodontal disease and either gender or disease extent. However, consistent with previous reports, a significant association was observed between smoking habits and periodontal disease (*p* = 0.009, with a higher prevalence of periodontitis among smokers. Duration of T1DM also influenced periodontal status (*p* = 0.048), with longer disease duration linked to an increased number of periodontitis cases. Finally, the absence of diabetes-related comorbidities—such as hypertension, nephropathy, neuropathy, retinopathy, diabetic foot, amputation, peripheral vascular disease, and coronary disease—was significantly associated with periodontal disease (*p* = 0.03). Among all the quantitative variables routinely assessed in T1DM patients, significant differences were observed in systolic blood pressure (*p* = 0.005) and HbA1c levels (*p* = 0.02), with both parameters being higher in T1DM patients with periodontitis compared to those with gingivitis or healthy periodontal status (**Table 3**). Altogether, these findings suggest that periodontal disease severity in individuals with type I diabetes is significantly influenced by lifestyle factors, diabetes management strategies, comorbidity profiles, and clinical indicators such as poor glycemic control and elevated blood pressure.

**Table 2:** Clinical characterization of diabetic patients across periodontal disease.

Variable		Periodontal disease			Statistical analysis		
		Healthy (n=11)	Gingivitis (n=33)	Periodontitis (n=51)	Statistic	*p* value	Effect Size*
Gender, n (%)	Male	7 (7)	17 (18)	38 (40)	4.67	*p* = 0.09	0.22
	Female	4 (4)	16 (17)	13 (14)			
Disease extension, n (%)	Localized	2 (2)	9 (11)	11 (13)	2.36	*p* = 0.12	0.17
	Generalized	31 (37)	42 (50)	73 (87)			
Smoker n (%)	Non smoker	11 (12)	30 (32)	35 (37)	9.33	*p* = 0.009	0.31
	Smoker	0	3 (3)	16 (17)			
Disease length, n (%)	< 1 year	0	2 (2)	2 (2)	15.3	*p* = 0.048	0.28
	1–5 years	3 (3)	2 (2)	5 (10)			
	6–10 years	4 (4)	2 (2)	10 (11)			
	11–20 years	4 (4)	16 (17)	15 (16)			
	>20 years	0	11 (12)	19 (20)			
Infusion pump, n (%)	No	5 (5)	15 (16)	41 (43)	12.5	*p* = 0.002	0.36
	Yes	6 (6)	18 (19)	10 (11)			
Comorbidities**, n, (%)	No	11 (12)	27 (28)	33 (35)	7.13	*p* = 0.026	0.28
	Yes	0	6 (6)	18 (19)			

**One of the following comorbidities: high blood pressure, nephropathy, neuropathy, retinopathy, diabetic foot, amputation, peripheral disease or coronary disease.

**Table 3:** Laboratory determinations commonly used to follow diabetes patients.

Variable	Periodontal disease			Statistical analysis*	
	Healthy (n=11)	Gingivitis (n=33)	Periodontitis (n=51)	Statistic	*p* value
Systolic blood pressure (mm/hg)	119 (23.8)	112 (24.25)	133 (18.5)	10.7	0.005
Diastolic blood pressure (mm/hg)	75 (5)	74 (11.5)	79 (12)	3.88	0.14
Cardiac frequency (bpm)	81 (19)	75 (16)	84 (15)	1.03	0.60
Weight	68 (20)	74 (17)	75 (20)	0.92	0.63
Height	171 (14)	170 (8)	169 (10)	2.60	0.27
Body mass index	24 (8)	24 (7)	25 (6)	0.21	0.91
Glucose	182 (58)	153 (119)	188 (104)	1.70	0.43
Fasting glycemia	170 (104)	180 (92)	174 (83)	3.06	0.21
Microalbuminuria	17 (44)	14 (83)	17 (54)	0.23	0.89
Cholesterol	153 (29)	159 (74)	168 (74)	0.22	0.90
HDL	51 (20)	56 (19)	59 (16)	0.49	0.78
LDL	96 (31)	92 (48)	94 (54)	0.36	0.84
Triglycerides	61 (65)	70 (50)	85 (68)	4.35	0.11
ESR	10 (9.25)	6 (9)	10 (7)	0.43	0.81
Creatinine	1.1 (0.19)	0.8 (0.14)	0.9 (0.24)	5.36	0.07
HbA1c	6.9 (1.35)	8.2 (1.35)	8.0 (1.10)	7.93	0.02

*For each variable, Kruskal-Wallis test was employed; statistical of test and *p*-value are reported. Data is present as median (Inter quartile range). HDL, high density lipoprotein, LDL, low density lipoprotein, ESR, erythrocyte sedimentation rate, HbA1c, glycated hemoglobin.

### Type I diabetes is associated with a worsen periodontal condition, even at equivalent levels of periodontal disease severity

**Figure 1:**
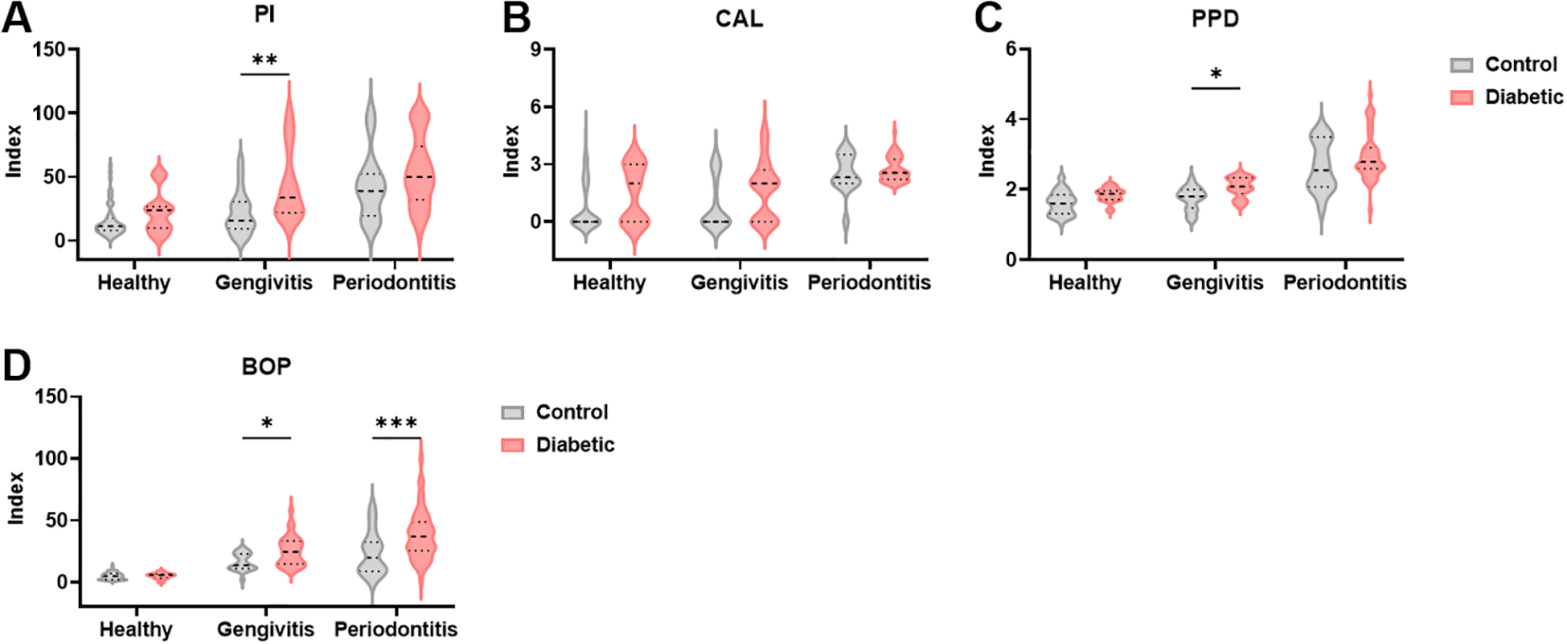
Clinical parameters used to evaluate periodontal disease are increased in diabetic patients. **(A–D)** Periodontal indices in healthy controls, individuals with gingivitis and periodontitis. PI, Plaque index, CAL, clinical attachment loss, PPD, pocket depth, BOP, Bleeding on probing. A two-way ANOVA with mixed-effect model was employed to compare control and diabetic individuals within each periodontal disease group, followed by a Dunnet’s multiple comparisons test. Results are presented as violin diagrams. *p<0.05; **p<0.01; ***p<0.001.

Consistent with the demographic findings, T1DM patients demonstrated elevated levels in all clinical parameters used to assess periodontal disease compared to non-diabetic controls with the same disease classification (**Figure 1**). For PI, a significant difference was found between T1DM and control individuals (F (1,184) = 1.24, *p* = 0.0008), among those with gingivitis (*p* = 0.002; **Figure 1A**). Regarding CAL, although a significant difference was found between groups overall (F (1,184) = 7.08, *p* = 0.008), no significant differences were observed within each specific disease category (**Figure 1B**). PPD levels were also significantly higher in T1DMdiabetic patients (F (1,184) = 11.9, *p* = 0.0007), also with significant differences noted among gingivitis patients (*p* = 0.04; **Figure 1C**). Interestingly, BOP was significantly elevated in T1DM patients (F (1,184) = 12.7, *p* = 0.0005), with statistically significant differences observed in both gingivitis (*p* = 0.04) and periodontitis groups (*p* = 0.0006; **Figure 1D**). Overall, these findings demonstrate that type I diabetes is associated with more severe periodontal disease compared to non-diabetic individuals, even within the same disease classification.

### Type 1 diabetic patients display a dysregulated local immune profile in gingival crevicular fluid

**Figure 2:**
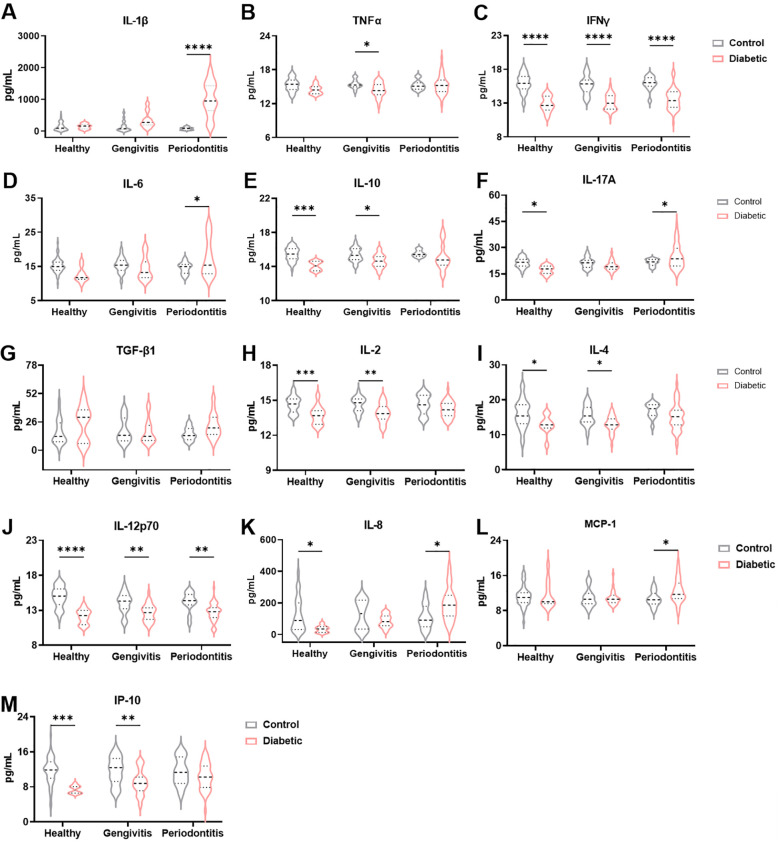
Immune profile in the gingival crevicular fluid between diabetic patients with periodontal disease and nondiabetic controls. Quantification of IL-1β **(A)**, TNFα **(B)**, IFNγ **(C)**, IL-6 **(D)**, IL-10 **(E)**, IL-17A **(F)**, TGF-β1 **(G)**, IL-2 **(H)**, IL-4 **(I)**, IL-12p70 **(J)**, IL-8 **(K)**, MCP-1 **(L)** and IP-10 **(M)** levels in gingival crevicular fluid. A two-way ANOVA with mixed-effect model was employed to compare control and diabetic individuals within each periodontal disease group, followed by a Dunnet’s multiple comparisons test. Results are presented as violin diagrams. *p<0.05; **p<0.01; ***p<0.001; ****p<0.0001.

Given the inflammatory nature of periodontitis and the well-established systemic inflammatory profile associated with T1DM, we investigated the cytokine levels in the gingival crevicular fluid (GCF) of our cohort (**Figure 2**). Levels of IL-1β were significantly elevated in T1DM individuals compared to non-diabetic controls (F (1, 166) = 41.90 and *p<*0.0001), with the most pronounced and statistically significant increase observed among those with periodontitis (*p<*0.0001), **Figure 2A**). In contrast, the pro-inflammatory cytokines TNFα and IFNγ were significantly decreased in T1DM patients relative to controls (F (1, 174) = 6.624 and *p* = 0.01 for TNFα and F (1, 178) = 114.4 and *p<*0.0001 for IFNγ; **Figures 2B, C**). Specifically, the reduction in TNFα was significant only in the gingivitis subgroup (*p* = 0.02), while IFNγ showed a consistent and highly significant decrease across all stages of periodontal disease (*p* < 0.0001 for all comparisons). A different pattern was observed for IL-6 (F (1, 166) = 0.1871 and *p =* 0.05): while levels were lower in T1DM individuals with healthy or gingivitis status, a significant increase was noted in those with periodontitis (*p* = 0.02, **Figure 2D**). The anti-inflammatory cytokine IL-10 was significantly reduced in T1DM individuals (F (1, 174) = 21.10 and *p* < 0.0001) across all periodontal disease classifications, presenting a significant decrease in healthy (*p* = 0.0003) and gingivitis groups (*p* = 0.03, **Figure 2E**). IL-17A, a cytokine with context-dependent pro- and anti-inflammatory functions, exhibited a nuanced expression pattern (F (1, 178) = 1.257, *p* = 0.04): decreased levels were observed in healthy and gingivitis diabetic individuals (*p* = 0.02 and *p* = 0.33, respectively), whereas a significant increase was detected in T1DM patients with periodontitis (*p* = 0.04, (**Figure 2F**). TGF-β1 levels showed a trend toward elevation in T1DM patients across all periodontal conditions; however, these differences did not reach statistical significance (F (1, 126) = 4.029 and *p* = 0.069; **Figure 2G**). Interestingly, despite the general pro-inflammatory milieu associated with diabetes, IL-2 levels were consistently reduced in T1DM patients compared to controls at all status of periodontal disease (F (1, 179) = 29.09 and *p* < 0.0001), with statistically significant decrease in healthy and gingivitis groups (*p* = 0.0005 and *p* = 0002, respectively, **Figure 2H**). This suggests that periodontitis in T1DM individuals may be predominantly driven by innate immune mechanisms. Supporting this hypothesis, levels of IL-4 and IL-12p70 — both associated with adaptive immune responses — were also decreased in T1DM patients across all periodontal stages (F (1, 172) = 15.04, and *p* = 0.0001 for IL-4 and F (1, 179) = 57.06 and *p* < 0.0001 for IL-12p70, **Figures 2I, J**). However, in IL-4 levels, a significant decrease was only observed in healthy and gingivitis (*p* = 0.04 and *p* = 0.03, respectively), while in IL-12p70 the decrease was significant in all periodontal disease stages (*p* < 0.0001 for heathy, *p* = 0.002 for gingivitis, and *p* = 0.002 for periodontitis). Additionally, we quantified three chemokines: IL-8 that recruits neutrophils, MCP-1, which recruits monocytes, and IP-10, which recruits T cells. IL-8 levels were significantly reduced in healthy and gingivitis-stage t1DM individuals compared to their non-diabetic counterparts (*p* = 0.03 and *p* = 0.21, respectively), yet significantly elevated in T1DM patients with periodontitis (*p* = 0.02, **Figure 2K**). MCP-1 levels tended to increase in diabetic patients with gingivitis and were significantly elevated in those with periodontitis (*p* = 0.04, **Figure 2L**). Conversely, although IP-10 levels were reduced in diabetic patients across all stages of periodontal disease (F (1, 159) = 21.58 and *p* < 0.0001), this reduction reached statistical significance only in healthy and gingivitis individuals (*p* = 0.0005 and *p* = 0.008, respectively; **Figure 2M**). Given the importance of HbA1c in monitoring patients with T1DM, we hypothesized that HbA1c levels could be associated with the cytokine profile. To test this, patients were stratified into two groups according to HbA1c values (<7% and >7%). No significant differences in cytokine levels were observed between these groups (data not shown). In addition, correlation analyses between HbA1c levels and all cytokines were performed, but no statistically significant associations were found (data not shown). Collectively, these findings suggest that periodontitis in T1DM individuals may be predominantly orchestrated by the innate immune system, potentially contributing to the exacerbated disease severity observed in this population due to an impaired or insufficient local acquired immune response.

### The salivary cytokine profile recapitulates that of the gingival crevicular fluid and exhibits a strong direct correlation with its levels

**Figure 3:**
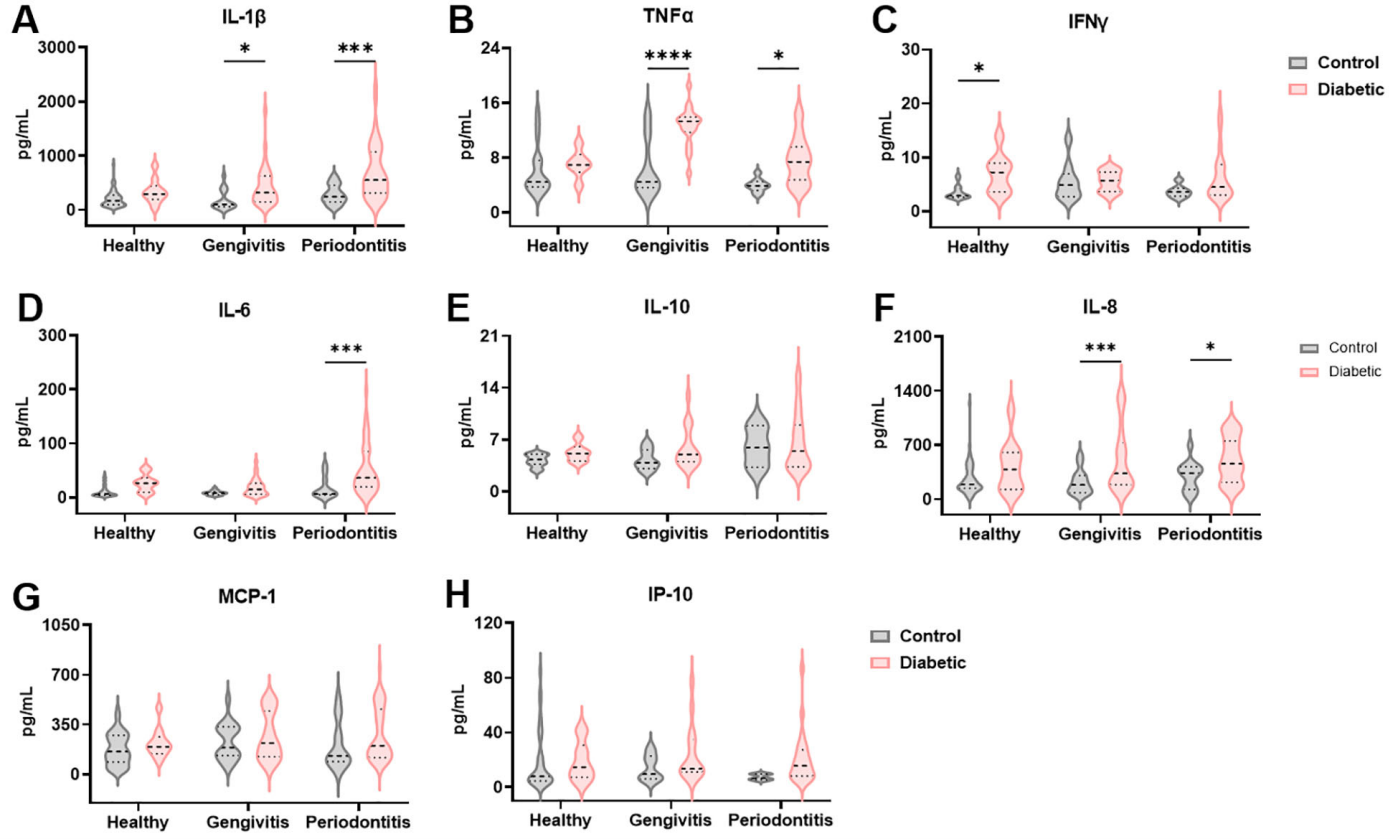
The inflammatory microenvironment in the gingival crevicular fluid of diabetic patients is reflected in the saliva. Quantification of IL-1β **(A)**, TNFα **(B)**, IFNγ **(C)**, IL-6 **(D)**, IL-10 **(E)**, IL-8 **(F)**, MCP-1 **(G)** and IP-10 **(H)** levels in saliva. A two-way ANOVA with mixed-effect model was employed to compare control and diabetic individuals within each periodontal disease group, followed by a Dunnet’s multiple comparisons test. Results are presented as violin diagrams. *p<0.05; ***p<0.001; ****p<0.0001.

To facilitate a less invasive profiling of periodontal disease, we also assessed the cytokine profile in saliva. As expected, not all cytokines previously identified in gingival crevicular fluid (GCF) were detectable in saliva. IL-1β, a classical pro-inflammatory cytokine associated with periodontal pathology, showed significantly elevated levels in T1DM individuals compared to non-diabetic controls (F (1, 176) = 20.46 and *p* < 0.0001), particularly in the gingivitis and periodontitis groups (*p* = 0.03 and *p* = 0.0001, respectively; **Figure 3A**). In contrast to GCF, salivary levels of TNFα and IFNγ were significantly increased in T1DM subjects (F (1, 97) = 22.75 and *p* < 0.0001 for TNFα and F (1, 76) = 8.092 and *p* = 0.006 for IFNγ; **Figures 3B, C**). TNFα was significantly elevated in t1DM patients with gingivitis (*p* < 0.0001) and periodontitis (*p* = 0.02), while IFNγ showed a significant increase only in healthy diabetic individuals (*p* = 0.02). IL-6 levels were again elevated in diabetic patients (F (1, 152) = 15.24, *p* = 0.0001), consistent with GCF findings, with significant differences observed in the periodontitis group (*p* = 0.0009, **Figure 3D**). IL-10 levels tended to be higher in healthy and gingivitis-stage diabetic individuals (F (1, 72) = 1.646, *p* = 0.20), and lower in those with periodontitis; however, these differences did not reach statistical significance (**Figure 3E**). Chemokine levels were also measured in saliva. IL-8 followed a pattern similar to that observed in GCF, showing increased levels in T1DM individuals with periodontal disease compared to non-diabetic controls (F (1, 173) = 20.05, *p* < 0.0001, **Figure 3F**). This increase was statistically significant in both the gingivitis (*p* = 0.0009) and periodontitis groups (*p* = 0.04). While both MCP-1 and IP-10 levels were also higher in diabetic patients, the differences did not achieve statistical significance (F (1, 175) = 4.138 and *p* = 0.05 for MCP-1; F (1, 95) = 3.200 and *p* = 0.08 for IP-10; **Figures 3G, H**). These results suggest that among salivary chemokines, IL-8 is the most promising marker for distinguishing periodontal status in T1DM individuals.

**Figure 4:**
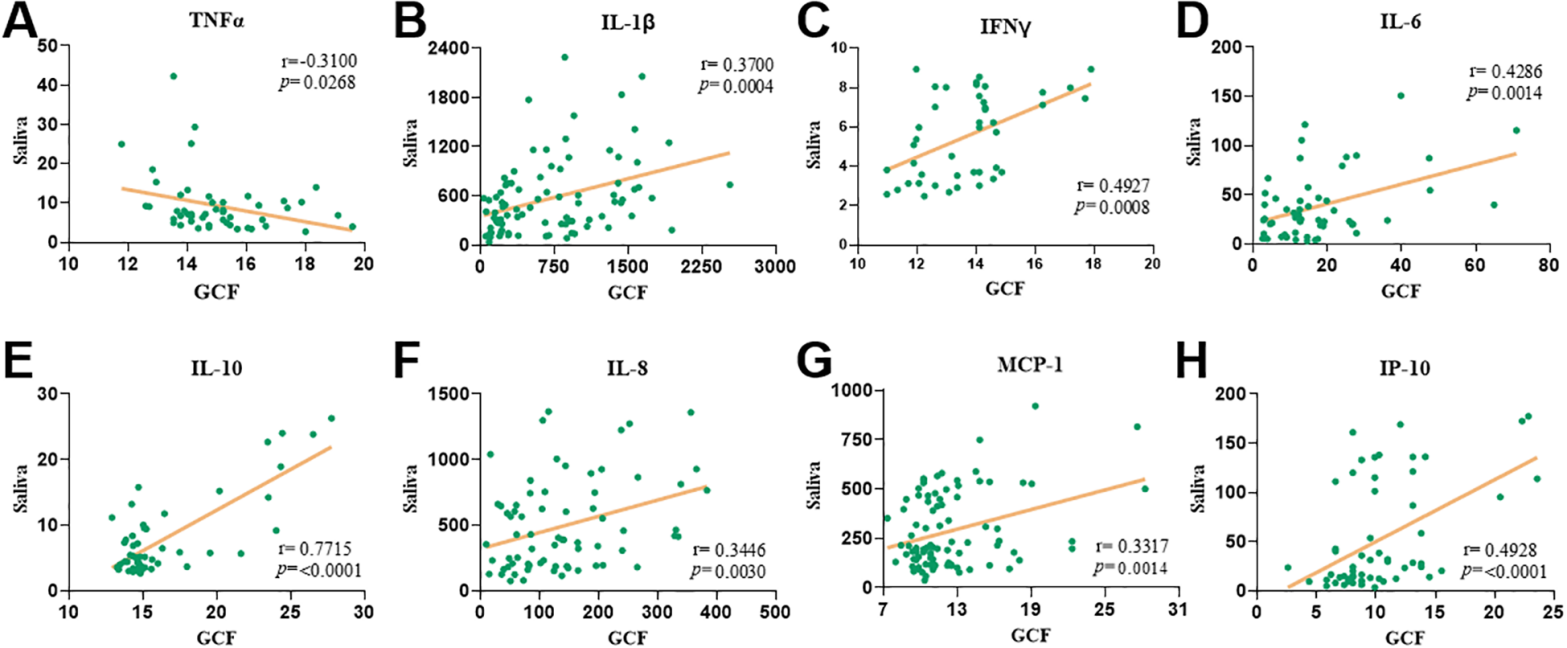
The levels of pro-inflammatory cytokines in gingival crevicular fluid correlate with saliva levels. Spearman’s correlations between gingival crevicular fluid and saliva levels of TNFα **(A)**, IL-1β **(B)**, IFNγ **(C)**, IL-6 **(D)**, IL-10 **(E)**, IL-8 **(F)**, MCP-1 **(G)** and IP-10 **(H)** levels. Both spearman’s correlation coefficient and p value are indicated in each correlation plot.

Given that disease diagnosis should aim to be as minimally invasive as possible, we next examined whether cytokine levels in GCF and saliva were correlated, to evaluate saliva’s potential as a diagnostic fluid in periodontal disease (**Figure 4**). Interestingly, TNFα levels showed a significant negative correlation between the two sample types (r = -0.31 and *p* = 0.03; **Figure 4A**). In contrast, IL-1β and IFNγ revealed significant positive correlations between GCF and saliva (r = 0.37 and *p* = 0.0004 for IL-1β; r = 0.49 and *p* = 0.0008 for IFNγ; **Figures 4B, C**). Additionally, IL-6 and IL-10 exhibited strong and significant positive correlations across the two fluids (r = 0.43 and *p* = 0.01 for IL-6 and r = 0.77 and *p* < 0.0001 for IL-10; **Figures 4D, E**). Similarly, the chemokines IL-8, MCP-1, and IP-10 demonstrated robust, statistically significant positive correlations between their levels in GCF and saliva (r = 0.34 and *p* = 0.003 for IL-8; r = 0.33 and *p* = 0.001 for MCP-1; r = 0.49 and *p* < 0.0001 for IP-10; **Figures 4F–H**). Altogether, these findings support the use of saliva as a non-invasive and reliable alternative to GCF for immunological profiling in patients with periodontal disease.

## Discussion

The past decades of research demonstrate that diabetes mellitus elevates the risk, severity, and progression of periodontal disease (23), a connection that is bidirectional, as persistent hyperglycemia alters inflammatory and immune responses in the periodontium (12, 14). Although research has focused mainly on type 2 diabetes, T1DM also doubles the risk of periodontitis and worsens outcomes yet remains less studied. To explore this relationship in T1DM, we conducted a case-control study stratifying participant by periodontal status and analyzed inflammatory cytokines in saliva and GCF. To characterize the clinical and inflammatory profiles of both conditions, along with the local immune responses, we quantified pro-inflammatory and anti-inflammatory cytokines in saliva and gingival crevicular fluid.

Our study found a significant association between T1DM and both the extent and severity of periodontal disease, with diabetic individuals showing a higher prevalence of generalized and severe periodontitis, while non-diabetic controls more frequently presented periodontal health. These findings align with previous studies reporting greater prevalence, severity, and progression of periodontitis in both T1DM (15, 18, 24, 25) and T2DM patients (26). While these findings support a strong association between T1DM and periodontitis, a direct causal relationship has not been stablished. T1DM results from autoimmune destruction of pancreatic β-cells, causing absolute insulin deficiency and sustained hyperglycemia. Key mechanisms include innate immune activation via Toll-like receptors, pro-inflammatory cytokines (IL-1, IL-6, TNFα, IL-17, IL-21), oxidative stress with ROS-driven activation of NF-κB/STAT1 pathways, and impaired immune checkpoints that fail to restrain autoreactive T-cell responses (27, 28). Type 2 Diabetes (T2DM) is a metabolic disorder characterized by insulin resistance and progressive β-cell dysfunction. Early stages feature hyperinsulinemia with reduced insulin signaling in muscle, liver, and adipose tissue (e.g., impaired GLUT4 translocation, glycogen synthesis). Chronic lipotoxicity, mitochondrial dysfunction, and ROS-mediated stress kinase activation (JNK, IKK) further disrupt signaling and accelerate β-cell exhaustion, culminating in overt hyperglycemia (29). Poor glycemic control also favors plaque accumulation and exacerbated gingival inflammation, further contributing to attachment loss (24).

No significant association was found between smoking status and T1DM in our cohort. However, most T1DM individuals were non-smokers with gingivitis or periodontitis, and periodontitis was significantly more prevalent among T1DM smokers (*p* < 0.009, **Table 2**). Smoking is a well-established risk factor for periodontitis, increasing its likelihood by approximately 85% (30). In T1DM patients, smoking has been associated with more severe periodontal manifestations (31) and, in long-term studies, to an increased risk of coronary heart disease when periodontal disease is also present (32). Interestingly, in our diabetic group, the absence of diabetes-related comorbidities was significantly associated with the presence of periodontal disease (*p* = 0.03). This unexpected finding may reflect the characteristics of a younger T1DM population, who have not yet developed systemic complications but already exhibit signs of periodontal inflammation. In contrast, existing literature consistently shows that poorer glycemic control and the presence of diabetes-related complications are associated with increased prevalence and severity of periodontitis in T1DM patients (33–35). In this same group of T1DM patients the values of systolic blood pressure (*p* = 0.005) and HbA1c levels (*p* = 0.02), were higher in periodontitis patients compared to those with gingivitis or healthy periodontal status. Although associations between T1DM and elevated systolic blood pressure remain inconsistent, likely due to variability in patient age and disease duration, several studies have consistently shown a robust link between periodontitis and hypertension, first noted by Brooks et al., 1999 (36) and later confirmed in larger cohorts, particularly for systolic pressure (37, 38).

Periodontal parameters, including PI, BOP, PPD, and CAL, were consistently higher in T1DM patients across all periodontal status categories, despite clinical matching with non-diabetic controls. Notably, BOP and PPD were significantly increased in T1DM individuals with gingivitis, indicating heightened gingival inflammation. In periodontitis, BOP remained significantly elevated in the diabetic group. Although CAL did not differ significantly, a trend toward greater attachment loss was observed in T1DM patients, possibly reflecting cumulative tissue damage influenced by individual variability, disease duration, and follow-up care. These findings reinforce the link between T1DM and increased periodontal inflammation and highlight the need for early periodontal monitoring in diabetic populations.

Previous studies in both children (19, 39–41) and adults (35, 42, 43) have emphasized the utility of periodontal assessments in characterizing the T1DM–periodontitis relationship. Building on this, our study is the first to evaluate cytokine profiles in both GCF and saliva across distinct periodontal statuses in T1DM patients. We identified a pro-inflammatory signature in GCF of T1DM individuals with periodontitis, marked by elevated IL-1β, IL-6, IL-8, and IL-17A, suggesting activation of both innate and adaptive immune pathways. Earlier work by Salvi et al. (1998, 2010) (44, 45) similarly demonstrated increased IL-1β, MMP-8, and MMP-9 levels in T1DM patients, independent of plaque or microbial differences, indicating an exaggerated host inflammatory response. Recent studies have highlighted the prognostic value of IL-6, IL-8, and IL-10 in distinguishing T1DM patients from healthy individuals (46). Similarly, a pro-inflammatory phenotype in T1DM patients with diabetic retinopathy was reported, marked by increased production of IL-6, IL-10, and IL-17A by myeloid cells, alongside reduced IFN-γ production by T cells (47). In our study, IFNγ levels were significantly decreased in the gingival crevicular fluid of T1DM patients compared to controls, independent of periodontal status, suggesting a systemic deficiency in adaptive immune function. This aligns with previous findings of reduced PBMC-derived IFNγ in T1DM with complications (47) and supports the role of IFNγ in the immunopathogenesis of T1DM by modulating both innate and adaptive immune pathways (48). We also found that TNFα was downregulated in T1DM patients, reaching statistical significance only in those with gingivitis. This contrasts with previous studies reporting marginally detectable TNFα levels in GCF of insulin-dependent diabetics, regardless of periodontal severity. However, diabetic monocytes exposed to *P. gingivalis* LPS secreted significantly more TNFα than non-diabetics, especially in severe periodontitis cases, suggesting a hyperinflammatory monocytic phenotype linked to disease severity (44). MCP-1 (CCL2), a chemokine involved in monocyte recruitment and amplification of local inflammation, was detected exclusively in the GCF of T1DM patients with periodontitis in our study (p < 0.05), supporting its role in periodontal immune responses (49). This may also suggest Th2 involvement via IL-4 promoter activation. Elevated systemic MCP-1 levels have been linked to diabetic complications and genetic susceptibility to T1DM (49). A meta-analysis confirmed higher MCP-1 levels in GCF of periodontitis patients compared to healthy controls (50), and another study showed that salivary MCP-1 and MIP-1α increase with disease severity, with MIP-1α being more sensitive in distinguishing gingivitis from healthy controls (51).

In our cohort, IL-8 levels in GCF were significantly higher in T1DM patients with periodontitis compared to non-diabetics (*p* < 0.05). Conversely, in healthy individuals, IL-8 was higher in non-diabetics than in T1DM patients, also with a significant difference. IL-8 is a key neutrophil chemoattractant involved in acute inflammation and has been studied across fluids including GCF, saliva, and serum (52). While most studies report elevated IL-8 in periodontitis and/or T1DM, findings remain partly inconsistent. For example, IL-8 was elevated in the saliva of diabetic children, regardless of periodontal status (39), and in plasma where it correlated with BMI (53). Other studies found no major differences, possibly due to mild disease or confounders (54). Still, meta-analyses generally support increased IL-8 in T1DM and/or periodontitis (52, 55, 56). Our study adds a novel perspective by analyzing IL-8 in both GCF and saliva from the same individuals. Salivary IL-8 was consistently higher in T1DM patients across all periodontal statuses, with significant differences in gingivitis and periodontitis, highlighting its potential as a biomarker.

IL-2, IL-4, and IL-12p70 levels were reduced in T1DM patients across all periodontal statuses compared to controls, suggesting that early immune responses may be dominated by innate cells (e.g., monocytes, neutrophils) rather than established Th1 polarization, possibly driven by IL-8 or MCP-1. Although CAL showed a slight increase in T1DM patients across periodontal groups, differences were not statistically significant. In contrast, IP-10 levels were consistently higher in non-diabetic controls, with significant differences in the healthy and gingivitis groups. IP-10, induced by IFNγ recruits monocytes, T cells, NK cells, and dendritic cells and has been implicated in periodontal tissue destruction (52).

Saliva is a non-invasive, easily accessible fluid that reflects both local and systemic changes, making it ideal for early disease detection. Its rich composition of inflammatory and immune biomarkers, along with low collection costs, supports its use in large-scale and point-of-care diagnostics (57). Building on its diagnostic potential, and considering the cytokine profiles observed in GCF, we extended our analysis to salivary cytokines as a less invasive method for periodontal disease profiling. As expected, several cytokines detected in GCF were undetectable in saliva, and those present appeared at lower concentrations, except for IL-6, IL-8, and MCP-1, which remained reliably detectable in both fluids. In saliva, IL-1β and IL-6 were significantly elevated in T1DM patients, especially those with periodontitis (*p* < 0.001), with IL-1β also distinguishing T1DM individuals with gingivitis (*p* < 0.05). These findings mirror GCF patterns and align with previous data showing IL-1β and IL-6 as key pro-inflammatory markers linked to periodontal disease severity but not progression (58). IL-1β remains one of the most promising salivary biomarkers for early periodontitis detection (59–61), although its association with T1DM is inconsistent (62, 63). Unlike in GCF, salivary TNFα and IFNγ were elevated in T1DM across all periodontal statuses, with significant increases in gingivitis (TNF-α, p < 0.0001) and healthy (IFNγ, *p* < 0.05) groups. IL-10 was detected but showed no significant association with T1DM, and existing literature on its role in periodontitis remains contradictory (58, 64, 65). IL-8 was significantly increased in diabetic individuals with gingivitis and periodontitis, aligning with GCF data, and emerged as the most promising salivary marker of periodontal status. MCP-1 and IP-10 were higher in diabetics, though not significantly. Strong positive correlations between gingival crevicular fluid and saliva were observed for IL-1β, IFNγ, IL-6, IL-10, IL-8, MCP-1, and IP-10, underscoring saliva’s value as a reliable, non-invasive medium for periodontal diagnostics. TNFα was the sole marker showing an inverse relationship between the two fluids. Overall, these results highlight the clinical utility of salivary immune profiling for periodontal disease assessment.

We acknowledge several limitations in our study, including the imbalance in the number of healthy, gingivitis, and periodontitis individuals within the non-diabetic control group compared to the T1DM group; the absence of HbA1c data; and the inability to determine glycemic control status (controlled vs. uncontrolled diabetes). Despite these limitations, our findings support a bidirectional relationship between T1DM and periodontal disease, which is more evident in individuals with periodontitis. The immune response is characterized by a predominant pro-inflammatory profile, notably involving IL-1β, IL-6, and IL-8. Among T1DM patients, IL-1β may help differentiate between gingivitis and periodontitis, while IL-8 appears to play a broader role in disease activity. Furthermore, the pro-inflammatory signature observed in GCF is reflected in the salivary cytokine profile, reinforcing the potential of saliva as a non-invasive tool for monitoring periodontal inflammation in T1DM individuals. Another limitation of our study is its cross-sectional design. While this type of approach provides valuable information about the disease stage at a specific point in time, the project would benefit from a longitudinal design, with multiple time points for the same patient, in order to establish a cause–effect relationship between disease stage and the observed cytokine profile.

## Data Availability

The original contributions presented in the study are included in the article/supplementary material. Further inquiries can be directed to the corresponding author.
